# Role of Alpha-Synuclein Protein Levels in Mitochondrial Morphology and Cell Survival in Cell Lines

**DOI:** 10.1371/journal.pone.0036377

**Published:** 2012-04-27

**Authors:** Min Zhu, Wenwei Li, Chuanzhen Lu

**Affiliations:** 1 Laboratory for Neurology of Institute of Integrative Medicine, Zhongshan Hospital, Fudan University Shanghai Medical College, Shanghai, P. R. China; 2 Institute of Neurology and Huashan Hospital, Fudan University Shanghai Medical College, Shanghai, P. R. China; 3 State Key Laboratory of Medical Neurobiology, Fudan University Shanghai Medical College, Shanghai, P. R. China; National Institute of Health, United States of America

## Abstract

α-Synuclein is highly associated with some neurodegeneration and malignancies. Overexpressing wild-type or mutant α-synuclein promotes neuronal death by mitochondrial dysfunction, the underlying mechanisms of which remain poorly defined. It was recently reported that α-synuclein expression could directly lead to mitochondrial fragmentation in vitro and in vivo, which may be due to α-synuclein localization on mitochondria. Here, we applied a double staining method to demonstrate mitochondrial morphogenetic changes in cells overexpressed with α-synuclein. We show that mitochondrial localization of α-synuclein was increased following its overexpression in three distinct cell lines, including HeLa, SH-SY5Y, and PC12 cells, but no alteration in mitochondrial morphology was detected. However, α-synuclein knockdown prevents MPP^+^-induced mitochondrial fragmentation in SH-SY5Y and PC12 cells. These data suggest that α-synuclein protein levels hardly affect mitochondrial morphology in normal cell lines, but may have some influence on that under certain environmental conditions.

## Introduction

α-Synuclein is a small and natively unfolded protein, and it is the first member of synuclein family which is named as earlier studies showed that α-synuclein only localizes in presynaptic terminals and nucleus [1]. Yet later, α-synuclein was shown in the somata of specific neuronal populations in the rat brain, e.g. in the SNpc, using a monoclonal antibody [2]. α-Synuclein has attracted considerable attention due to its involvement in neurodegenerative diseases, such as Parkinson's disease (PD) and Alzheimer's disease [3], [4], [5]. Beyond those, aberrant expression of α-synuclein may be highly associated with human malignancies [6], [7]. However, less is known about its normal function.

Several studies already noticed some connection between α-synuclein and 1-methyl-4-phenyl-1,2,3,6-tetrahydropyridine (MPTP), a well characterized neurotoxin for inducing PD models at present, which oxidized in vivo into the toxic substrate 1-methyl-4-phenylpyridinium (MPP^+^) [8], [9], [10]. Vila et al. found that MPTP enhances α-synuclein expression in vivo [11]; Dauer et al. reported that α-synuclein is required for MPTP-induced apoptosis as α-synuclein null mice presents striking resistance to MPTP [12]. These studies established a model to link environmental and genetic factors in PD-like cell death; still, the mechanism underlying is poorly elucidated.

Lately, a pathogenic link between α-synuclein and mitochondria was established by the observations that some transgenic mice overexpressing wild-type or mutant α-synuclein develop abnormal mitochondrial morphology [13], [14], [15]. Buttner et al. showed that depletion of mitochondria DNA in yeast inhibits ROS formation and cell apoptosis induced by α-synuclein [16], further suggesting a direct functional connection between α-synuclein and mitochondria. More than those, a series of articles indicates that α-synuclein may directly interact with mitochondria. We reported that, in addition to its predominantly cytosolic and vesicular localization, a fraction of α-synuclein localizes in the mitochondria under physiological condition [17], which is confirmed by many other studies [18], [19]. Nevertheless, the normal function and pathogenic role of mitochondrial α-synuclein need further investigation.

Recently, Kamp et al. reported that α-synuclein has an inhibitory function on membrane fusion, and it binds to mitochondria and directly leads to mitochondrial fragmentation when overexpressed in cell cultures and Caenorhabditis elegans [20]. In addition, Nakamura et al. described that the effect is not accompanied by changes in the morphology of other organelles, including endoplasmic reticulum (ER) and lysosomes [21]. This may reveal a novel model of mitochondrial dynamic regulation, yet some questions remains unanswered: Since recombinant α-synuclein induces fission of artificial membranes, why does it have no influence on other lipid membranes in cells, such as ER and lysosomes, except mitochondria? Therefore, more direct evidence is needed to show the role of α-synuclein levels in mitochondrial morphology.

Mitochondria and α-synuclein were double-stained in this study, which not only allows us to compare mitochondrial morphology and α-synuclein expression and distribution among three distinct cell lines, including Hela, SH-SY5Y and PC12 cells, but also to directly assess mitochondrial morphological changes in cells following α-synuclein overexpression. Our results indicate that α-synuclein expression levels has little influence on mitochondrial morphology in normal cells, but knockdown of α-synuclein prevents MPP^+^-induced mitochondrial fragmentation in SH-SY5Y and PC12 cells. These data imply that α-synuclein plays no role in mitochondrial morphology under normal condition, but may have some effect on that at the presence of certain environment factors.

## Results

### Mitochondrial Morphological Characteristics in Cell Lines Are Hardly Affected by Fixation

To observe the effects of α-synuclein overexpression on mitochondrial morphology, we need to track down α-synuclein overexpressed cells. However, some traditional approaches may be unsuitable for α-synuclein, such as fluorescent protein tags used in many papers. As a typical natively unfolded protein, α-synuclein lacks of rigid and ordered structure under physiological conditions in vitro [22], [23], and its structure depends extremely on its environment and accommodates a number of unrelated conformations, which might affect its functions accordingly [24]. If α-synuclein was tagged with a fluorescent protein, whose molecular weight is even bigger than α-synuclein, we could not guarantee that this would not affect its structure or functions. Similar problems may remain when a fluorescent protein is co-transfected as an independent reporter. Another option is to transfect cells with untagged full length human *SNCA* cDNA, stain live cells with MitoTracker, fix them and then process for immunofluorescence staining of α-synuclein, since MitoTracker Red CMXRos stains mitochondria in live cells and is well-retained in fixed, permeabilized cells.

**Figure 1:**
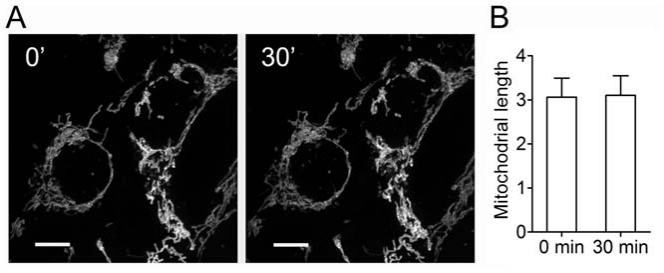
Cell Fixation Does not Alter Mitochondrial Morphology Probed by Staining with MitoTracker-Red CMXRos. **A.** Representative images of mitochondria in the same SH-SY5Y cells stained with MitoTracker-Red CMXRos were taken immediately before and 30 min after fixation with 4% paraformaldehyde on live cell imaging system. Mitochondrial morphology in SH-SY5Y cells remains almost the same after fixation for 30 min. Scale bars for 10 µm. **B.** Quantitative analysis of changes in mitochondrial length of the same SH-SY5Y cells in images taken immediately before and 30 min after fixation. The experiment was carried out five times, and no perceptible difference in mitochondrial length is detected in SH-SY5Y cells after fixation.

Mitochondrial length (or aspect ratio) is usually used to assess mitochondrial morphology in live cells [25], [26], [27], thus firstly, we need to test whether mitochondrial length would be affected by cell fixation. SH-SY5Y, PC12 or Hela cells were stained with MitoTracker-Red CMXRos and then observed on live cell imaging system using a 100× objective. Images of the same cells were taken immediately before and 30 min after fixation with 4% paraformaldehyde to detect mitochondrial morphogenetic alterations, one image often covers 1–2 SH-SY5Y or Hela cells or 4–8 PC12 cells, and the experiment was carried out five times for each cell line. As shown in Figure 1, mitochondrial length in SH-SY5Y cells remained almost the same after fixation for 30 min, and no excessive mitochondrial fragmentation was observed, suggesting that mitochondrial morphology in live cells is well reserved by fixation. Similar results were seen in other two cell lines.

### Different Patterns of α-Synuclein Distribution Were Observed among Cell Lines

**Figure 2:**
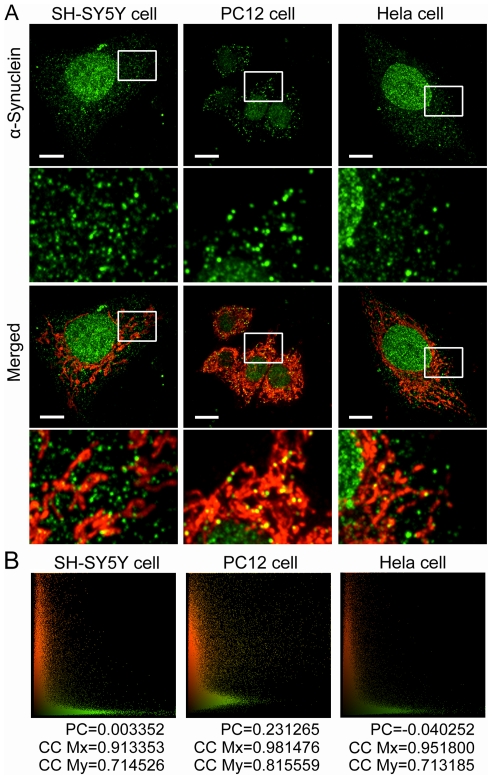
α-Synuclein Distribution Patterns and Mitochondrial Morphology in Three Cell Lines. **A.** Representative double-staining images show the patterns of α-synuclein distribution (green) and mitochondrial morphology (red) in SH-SY5Y, PC12 and Hela cells. Low levels of α-synuclein were expressed in all three cell lines, and it distributes in a small dotted fashion. α-Synuclein is relatively highly expressed in the nucleus in SH-SY5Y and Hela cells other than in PC12 cells. Amplified images further demonstrate that only a small part of cytoplasmic immunofluorescent staining of α-synuclein co-localizes with mitochondria in SH-SY5Y and Hela cells, while most of cytoplasmic α-synuclein reside in mitochondria in PC12 cells. Scale bars for 10 µm. **B.** Cytofluorogram analysis of the double-staining images above shows that Pearson's Correlation (PC) coefficient is much higher in PC12 cells than those in SH-SY5Y and Hela cells. CC, colocalization coefficient.

Since mitochondrial morphological characteristics are barely affected by cell fixation, we further processed SH-SY5Y, PC12 or Hela cells for α-synuclein immunofluorescence after MitoTracker staining, which would allow us to compare endogenous α-synuclein expression and distribution among cell lines and assess mitochondrial morphology at the same time. As shown in Figure 2A, low levels of α-synuclein were expressed in all three cell lines, and it does not distribute evenly in cells but in a small dotted fashion. α-Synuclein distributions were quite different among cell lines. α-Synuclein was relatively highly enriched in the nucleus in Hela and SH-SY5Y cells other than in PC12 cells. Punctate staining of α-synuclein spreaded all over the cytoplasm of SH-SY5Y and Hela cells, while it was far less dispersed in the cytoplasm of PC12 cells. Further amplified images demonstrated clearly that most of cytoplasmic α-synuclein co-localized with mitochondria in PC12 cells, whereas only a small part of cytoplasmic α-synuclein reside in mitochondria in SH-SY5Y and Hela cells. That was also confirmed by cytofluorogram analysis in Figure 2B, showing that Pearson's Correlation (PC) coefficient in PC12 cells was much higher than those in SH-SY5Y and Hela cells. The results above suggest that most of cytoplasmic α-synuclein accumulates in mitochondria in PC12 cells. Up to date, we have not found a similar report in other articles. Since PC12 cell line is from rat pheochromocytoma, whether the peculiar distribution of α-synuclein plays a key role in pathogenesis of pheochromocytoma requires further study. However, it provides a good model to investigate the effects of mitochondria localization of α-synuclein on mitochondrial morphology, but yet no excessive mitochondrial fragmentation was observed in PC12 cells when compared with SH-SY5Y and Hela cells.

### α-Synuclein Overexpression does not Directly Influence Mitochondrial Morphology in Cell Lines

To investigate whether α-synuclein overexpression leads to mitochondrial fragmentation, SH-SY5Y, PC12 and Hela cells were transfected with a constructed plasmid expressing human α-synuclein (SNCA) using FuGENE HD Transfection Reagent. Firstly, different ratios of plasmid DNA (µg)/transfection reagent (µl) (1∶3, 1∶4, 1∶5, 1∶6) were applied to optimize transfection efficiency for each cell line. Immunoblotting analysis after transfection for 48 h showed that 1 µg plasmid DNA/4 µl transfection reagent already provided the best transfection efficiency for SH-SY5Y cells (Figure S1), which was then used in the following experiments. Similar results were seen in PC12 and Hela cells (data not shown).

**Figure 3:**
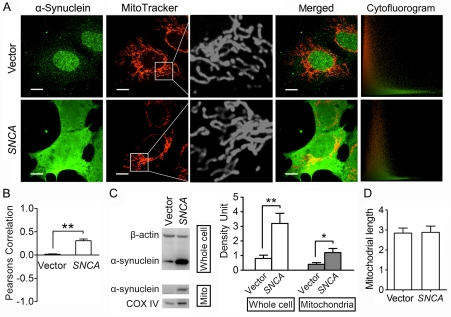
α-Synuclein overexpression promotes its mitochondrial localization without inducing mitochondrial fragmentation in SH-SY5Y cells. **A.** Representative double-staining images of α-synuclein (Green) and mitochondria (Red) in SH-SY5Y cells overexpressed with α-synuclein (*SNCA* group) or transfected with an empty vector (Vector group) for 48 h. Amplified images further show that no obvious alteration in mitochondrial morphology between these two groups. Cytofluorogram analysis demonstrates that PC coefficient is remarkably elevated in α-synuclein overexpressed cells (PC = 0.313104, CC Mx = 0.840827, CC My = 0.986122) compared with Vector group (PC = 0.021315, CC Mx = 0.964957, CC My = 0.780582). Scale bars for 10 µm. B. Quantitative analysis demonstrates that PC coefficients are remarkably elevated in *SNCA* group compared with Vector group. C. Immunoblotting assay shows that α-synuclein expression levels are significantly elevated in SH-SY5Y cell lysates and mitochondrial lysates after transfected with *SNCA* for 48 h (n = 4). **D.** Quantitative analysis of changes in mitochondrial length shows no significant statistic difference between *SNCA* group and Vector group. Images of 20 cells from each group were processed for cytofluorogram (B) and mitochondrial morphology analysis (D), and the experiment was repeated three times. *P<0.05, **P<0.01.

**Figure 4:**
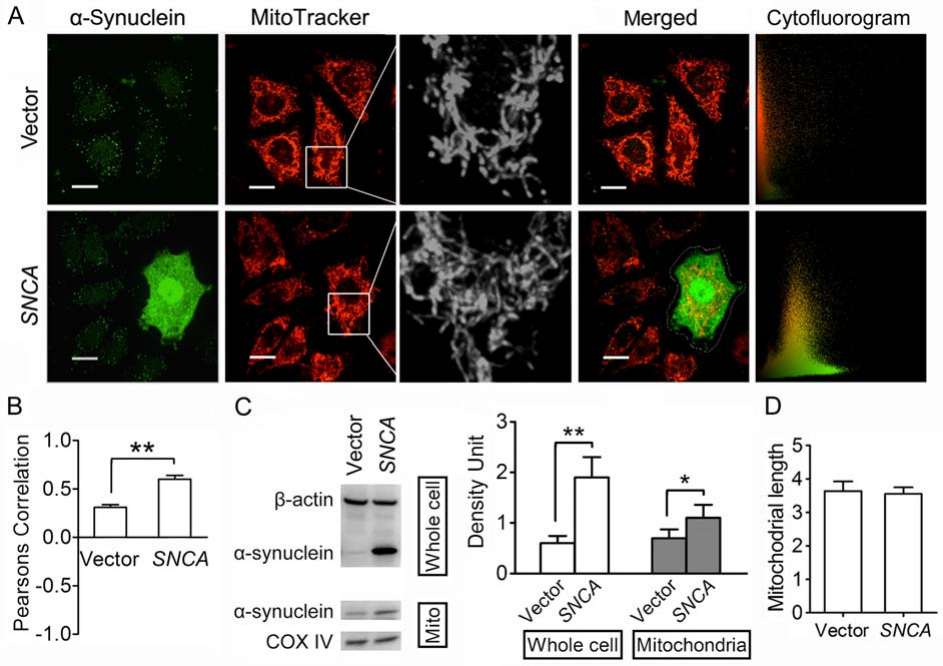
α-Synuclein overexpression increases its localization on mitochondria without causing mitochondrial fragmentation in PC12 cells. **A.** Representative double-staining images of α-synuclein (Green) and mitochondria (Red) in PC12 cells transfected with *SNCA* or an empty vector for 48 h. Amplified images clearly demonstrate that no perceptible alteration in mitochondrial morphology between these two groups. Cytofluorogram analysis shows that PC coefficient of that α-synuclein overexpressed cell (PC = 0.574982, CC Mx = 0.972038, CC My = 0.98707) is much higher than Vector group (PC = 0.316512, CC Mx = 0.962559, CC My = 0.439231). Scale bars for 10 µm. B. Quantitative analysis shows that PC coefficients in *SNCA* group are remarkably elevated compared with Vector group. C. Immunoblotting assay shows that α-synuclein expression levels in PC12 cell lysates and mitochondrial lysates are both significantly increased after *SNCA* transfection for 48 h (n = 4). **D.** Quantitative analysis of changes in mitochondrial length shows no significant statistic difference between *SNCA* group and Vector group. Images of 20 cells from each group were processed for cytofluorogram (B) and mitochondrial morphology analysis (D), and the experiment was repeated three times. *P<0.05, **P<0.01.

**Figure 5:**
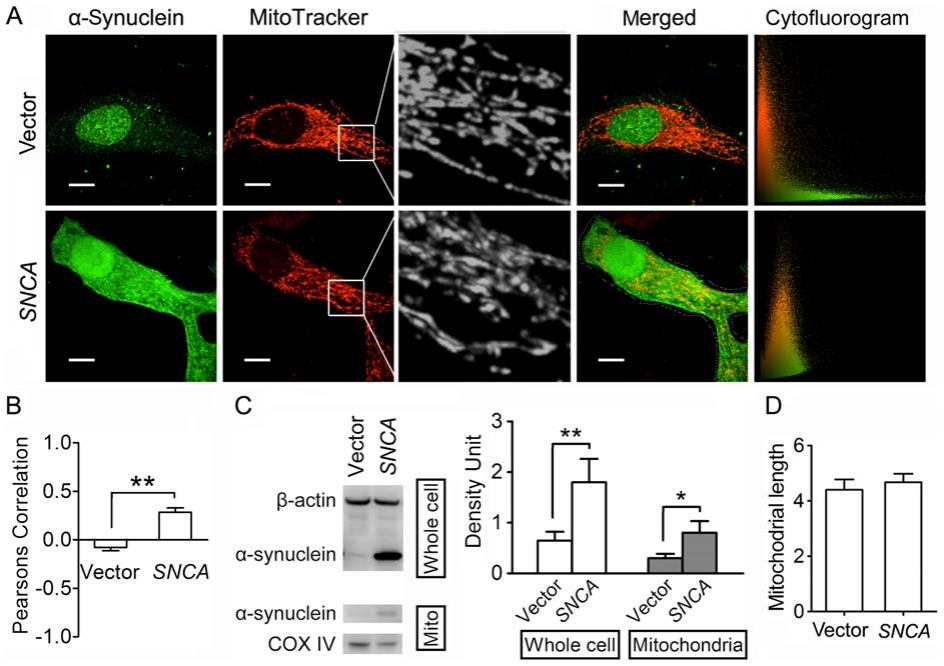
α-Synuclein overexpression in Hela cells promotes its co-localization with mitochondria without affecting mitochondrial morphology. **A.** Representative double-staining images of α-synuclein (Green) and mitochondria (Red) in Hela cells transfected with *SNCA* or an empty vector for 48 h. Further amplified images show that no obvious change in mitochondrial morphology between these two groups. Cytofluorogram analysis demonstrates that PC coefficient in that α-synuclein overexpressed cell is remarkably elevated (PC = 0.374221, CC Mx = 0.998574, CC My = 0.845116) compared with Vector group (PC = −0.0338751, CC Mx = 0.905173, CC My = 0.796768). Scale bars for 10 µm. B. Quantitative analysis demonstrates that PC coefficients in *SNCA* group are much higher than Vector group. C. Immunoblotting assay demonstrates that α-synuclein expression levels in Hela cell lysates and mitochondrial lysates are both remarkably increased after *SNCA* transfection for 48 h (n = 4). **D.** Quantitative analysis of changes in mitochondrial length shows no significant statistic difference between *SNCA* group and Vector group. Images of 20 cells from each group were processed for cytofluorogram (B) and mitochondrial morphology analysis (D), and the experiment was repeated three times. *P<0.05, **P<0.01.

After transfection for 48 h, cells overexpressed with α-synuclein or transfected with an empty vector were both processed for double-staining of mitochondria and α-synuclein, and then observed on live cell imaging system using a 100× objective. Images of 20 cells from random view fields were taken from each group and processed for mitochondrial morphology and cytofluorogram analysis, and the experiment was repeated three times. The expression of α-synuclein was also assayed by immunoblotting. As shown in Figure 3A and C, SH-SY5Y cells transfected with human *SNCA* presented a high level of α-synuclein expression, compared with a pretty low level in the cells transfected with an empty vector. Representative mitochondrial images demonstrated no remarkable mitochondrial fragmentation in α-synuclein overexpressed cells, compared with those in the cells transfected with an empty vector (Figure 3A). And quantitative analysis of mitochondrial length also showed no significant difference between these two groups of cells (vector group: 2.85±0.25 vs. *SNCA* group: 2.88±0.31, P>0.05) (Figure 3D). Similar results were detected when we observed the effects of α-synuclein overexpression on mitochondrial morphology in PC12 (vector group: 3.64±0.29 vs. *SNCA* group: 3.55±0.20, P>0.05) (Figure 4A and D) and Hela cells (vector group: 4.41±0.37 vs. *SNCA* group: 4.68±0.30, P>0.05) (Figure 5A and D). These data suggest that it may be a common biological event that simply raising α-synuclein expression levels would not affect mitochondrial morphology directly.

### α-Synuclein Overexpression Promotes Its Mitochondrial Localization without Inducing Mitochondrial Fragmentation

It is suggested that the mitochondrial fragmentation induced by α-synuclein overexpression may be due to its localization on mitochondria [20]. To examine whether α-synuclein localization on mitochondria was promoted after *SNCA* transfection in our experiment, we isolated mitochondria and analyzed mitochondrial α-synuclein expression by immunoblotting. Mitochondrial or cytosolic extracts were first examined by immunoblotting for the presence of cytochrome oxidase subunit IV (COX IV) and β-actin. A robust band of COX IV was detected from the mitochondrial extracts, while the β-actin band was pretty weak; they were opposite in the cytosolic fraction, suggesting that the mitochondrial fraction was enriched with mitochondria, while few mitochondria were detected in the cytosolic fraction (Figure S2). α-Synuclein expression in mitochondrial pellets was very low in SH-SY5Y (Figure 3C), PC12 (Figure 4C) and Hela cells (Figure 5C), yet it was remarkably increased in all three cell lines after *SNCA* transfection for 48 h.

Representative double-staining images of mitochondria and α-synuclein demonstrated that after *SNCA* transfection for 48 h, α-synuclein expression in all three cell lines was significantly increased and its distribution was greatly changed compared with those in the cells transfected with an empty vector. α-Synuclein was highly enriched in the nucleus in Hela and PC12 cells after *SNCA* transfection (Figure 4A and 5A), and its expression was also strong in the nucleus in SH-SY5Y cells, but even stronger in the cytoplasm (Figure 3A). α-Synuclein was expressed all over the cytoplasm in PC12 cells, where some peculiar punctate staining of α-synuclein was more standout and mostly co-localized with mitochondria, as shown in the amplified images in Figure S3. Cytofluorogram analysis of those double-staining images demonstrated that in all three cell lines, PC coefficients in α-synuclein overexpressed cells were magnificently elevated compared with the cells transfected with an empty vector (Figure 3A and B, 4A and B, 5A and B). These indicate that more α-synuclein co-localizes with mitochondria in α-synuclein overexpressed cells.

Although mitochondrial localization of α-synuclein was increased following *SNCA* transfection, mitochondria in three cell lines still remain an elongated tubular and thread-like shape and no significant change in mitochondrial length was detected between α-synuclein overexpressed cells and those transfected with an empty vector (Figure 3A and D, 4A and D, 5A and D). This indicates that α-synuclein overexpression increases its localization on mitochondria without affecting mitochondrial morphology in cell lines.

### α-Synuclein Suppression does not Alter Mitochondrial Morphology, but Prevents Mitochondrial Fragmentation in Dopaminergic Cell lines Exposed to MPP^+^

**Figure 6:**
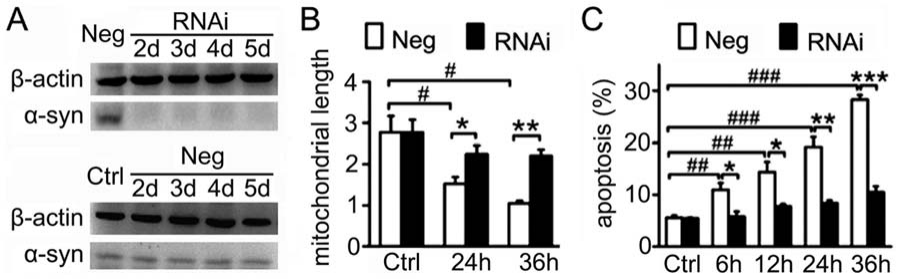
α-Synuclein knockdown prevents MPP^+^-induced cell apoptosis and mitochondrial fragmentation in SH-SY5Y cells. **A.** Immunoblotting assay demonstrates that α-synuclein expression is remarkably suppressed in SH-SY5Y cells transfected with *SNCA* siRNA (RNAi group) for 2–5 d, yet it is hardly affected in cells transfected with a negative control sequence (Neg group) (n = 5). **B.** Quantitative analysis of changes in mitochondrial length shows that MPP^+^ (1 mM) decreases mitochondrial length in Neg group, however, it has little effect on the index in RNAi group. Images of 20 cells from each group were processed for mitochondrial morphology analysis, and the experiment was repeated three times. **C.** Flow cytometric analysis of cell apoptosis shows that MPP^+^ leads to severe cell injury in Neg group, while it slightly harms SH-SY5Y cells in RNAi group (n = 4). *P<0.05, **P<0.01, ***P<0.001 Neg versus RNAi; #P<0.05, ##P<0.01, ###P<0.001 compared with Neg control.

**Figure 7:**
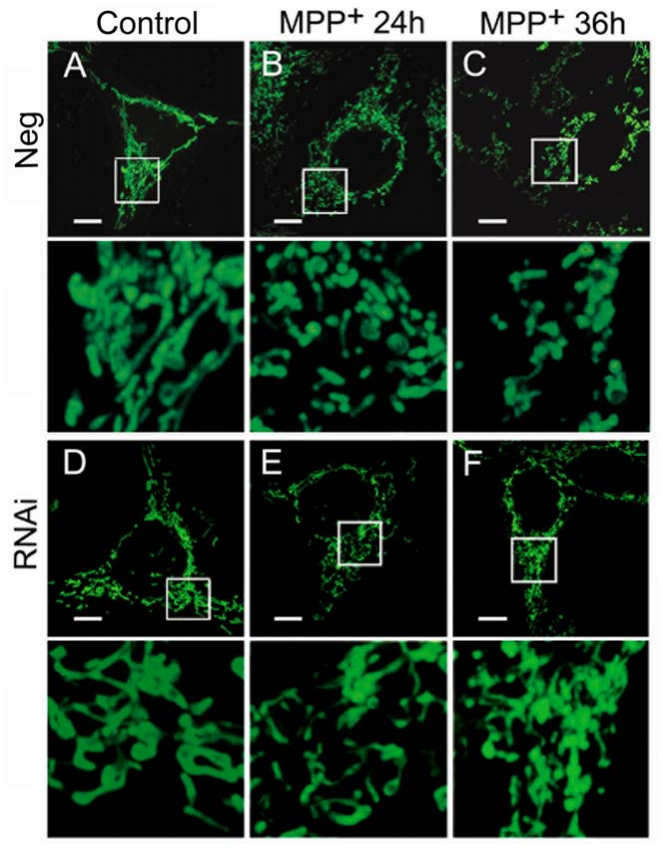
The effects of α-synuclein suppression on mitochondrial morphology in SH-SY5Y cells. Representative images taken by live cell imaging system show no obvious alteration in mitochondrial morphology between Neg group (A) and RNAi group (D). MPP^+^ (1 mM) induces severe mitochondrial fragmentation in Neg group (B and C) but has little effect on mitochondrial morphology in RNAi group (E and F). Scale bar for 10 µm.

Since α-synuclein overexpression has no effect on mitochondrial morphology, we next detected the effects of α-synuclein knockdown on mitochondrial morphology. SH-SY5Y or PC12 cells were transfected with small interfering RNA (siRNA) of *SNCA* or a negative control sequence, and the expression of α-synuclein was assayed by immunoblotting. *SNCA* siRNA provided good knockdown of α-synuclein, while negative control sequence hardly affected α-synuclein expression in SH-SY5Y (Figure 6A) or PC12 cells (Figure S4B)after transfection for 2 to 5 d. Mitochondria were visualized by staining with MitoTracker-Green FM, and images were taken and analyzed and mitochondrial length was quantified as described above. There was no perceptible difference in mitochondrial length between negative group and RNAi group of SH-SY5Y (Figure 6B and 7) or PC12 cells (Figure S4A and D), implying that α-synuclein suppression does not affect mitochondrial morphology in those two dopaminergic cell lines, either.

Recently, Wang et al. observed mitochondrial fragmentation in SH-SY5Y cells induced by MPP^+^, a dopaminergic neurotoxin [28]. To probe whether α-synuclein is involved in MPP^+^-induced mitochondrial fragmentation, cell cultures from both groups above were treated with 1 mM MPP^+^ after transfection for 2 d. After MPP^+^ exposure for 24 h and 36 h, mitochondrial morphology were determined as described above, and apoptotic cells were determined by flow cytometry. As demonstrated in Figure 6C, MPP^+^ treatment resulted in severe cell damage in the negative group of SH-SY5Y cells, and the percentage of apoptotic cells reached 19.23±1.90% at 24 h and 28.31±0.93% at 36 h. Nonetheless, cellular injury was prevented remarkably in the RNAi group of SH-SY5Y cells, where the percentage of apoptotic cells never reached more that 15% after MPP+ exposure. MPP^+^ treatment also led to remarkable mitochondrial fragmentation in the negative group of SH-SY5Y cells (Figure 7A–C), and mitochondrial length was significantly decreased by MPP^+^ (Figure 6B). However, severe mitochondrial fragmentation was hardly seen in the RNAi group of SH-SY5Y cells after MPP^+^ exposure (Figure 7D–F), and mitochondrial length was much longer compared with the negative group (Figure 6B). Similar results were seen in PC12 cells, that α-synuclein suppression by RNAi attenuated MPP^+^-caused mitochondrial fragmentation (Figure S4A and D) and cell apoptosis (Figure S4C). These indicate that α-synuclein may not contribute much to retain normal mitochondrial morphology, yet its suppression resists MPP^+^-induced mitochondrial fragmentation in dopaminergic cell lines.

## Discussion

Mitochondria undergo continuous fission and fusion in living cells, which is necessary for maintaining mitochondrial network morphology and physiological functions. Mitochondrial fission and fusion were first described in yeast [29], and found that it is mediated by a group of large GTPases [30], [31], [32], [33]. Although their precise mechanism of action is unclear, the conservation of these GTPases across species suggests that there are some similar mechanisms of mitochondrial dynamics between mammalian cells and yeast [34]. In addition to these GTPases, other proteins have been implicated in the regulation of mitochondrial fusion and fission in mammalian cells, however, almost all of them participate in the regulation of mitochondrial dynamics by interacting with these GTPases. For example, mitochondria-associated PTEN-induced kinase 1 (PINK1), a protein linked to familial PD, regulates mitochondrial dynamics through interaction with the fission/fusion machinery [35]. Owing to lack of the structural basis, α-synuclein may not be able to direct mitochondrial fission or fusion. Although Kamp et al. described that α-synuclein could inhibit protein-free model membrane fusion in *vitro*, but the concentration of α-synuclein in these reactions was much higher than in normal cells. Therefore, it is difficult to rule out a possibility that it may be the result of a simple physical action.

α-Synuclein protein is specific to the vertebrate and expresses at high levels in the brain, especially within developing neurons [36], suggesting its roles in the development of central nervous system. Supposing that α-synuclein directly regulates mitochondrial dynamics, mitochondrial fission/fusion machinery in vertebrate neurons would be very different from that in other types of cells in vertebrate or in non vertebrate neurons, yet there is no evidence to support this hypothesis. When cells become malignant, some of them, such as Hela cells, start to express α-synuclein while the normal cells would not [6], [7], nonetheless, no data indicate that excessive mitochondrial fission was found in the malignant cells. Additionally, mitochondria are shorter in immature cells than in mature cells [37], [38], and mitochondrial fission is essential for embryonic development. Mice lacking the mitochondrial fission GTPase Drp1 have developmental abnormalities and die after embryonic day 12.5 and neural cell-specific Drp1 null mice die shortly after birth [39]. Although α-synuclein expression levels are pretty higher in embryos or infants than in adults [40], [41], mice lacking α-synuclein show only subtle abnormalities in neurotransmission [42], [43], [44], lending an evidence to support that α-synuclein expression levels may not affect mitochondrial morphogenesis directly. Since suppression of α-synuclein has potential values for therapy of PD and related diseases, it is of great biological importance that α-synuclein would not play a key role in essential physiological functions.

In conclusion, our results demonstrate that neither α-synuclein expression levels nor its localization to mitochondria affects mitochondrial dynamics. But, this does not mean that α-synuclein is in no way involved in regulation of mitochondrial dynamics. In fact, we found that α-synuclein participates in MPP^+^ induced mitochondrial fragmentation, suggesting that environmental risk factors may be essential for α-synuclein to gain a function to be involved into mitochondrial dynamics, which required further studies.

Recently, genetic contributions in PD pathology have received much attention, but cross-sectional twin studies found that even within families affected by monogenic PD, the age of onset, symptoms and end-stage pathology may be quite variable [45], [46], [47]. More than that, no obvious connections were found in PD patients between α-synuclein expression and neuronal damage. In fact, α-synuclein protein levels are far higher during early human development than late when PD usually occurs [40], and α-synuclein mRNA label is strong in both affected and unaffected neurons in PD [48]. A possible explanation is that certain environmental exposure is necessary for wild-type α-synuclein to gain its toxicity in PD patients, and our results may lend evidences to support the hypothesis.

## Materials and Methods

### Cell culture

SH-SY5Y, Hela and PC12 cells were obtained from American Tissue Culture Collection and used within 20 passages of the original vial. SH-SY5Y cells were grown in Dulbecco's modified eagle's medium (DMEM)/F12 medium supplemented with 15% fetal bovine serum (FBS), 100 IU/ml penicillin, 0.1 mg/ml streptomycin and 3.7 g/L NaHCO_3_. Hela cells were grown in DMEM medium supplemented with 10% FBS, 100 IU/ml penicillin, 0.1 mg/ml streptomycin and 3.7 g/L NaHCO_3_. PC12 cells were grown in DMEM medium supplemented with 10% horse serum, 5% FBS, 100 IU/ml penicillin, 0.1 mg/ml streptomycin and 3.7 g/L NaHCO_3_. Cell cultures were all kept at 37°C in a saturated humidity air atmosphere containing 5% CO_2_.

### Transfection of *SNCA*

Cells at 50% confluency were transiently transfected using FuGENE HD Transfection Reagent (Roche) following the instruction of the manufacturer. Full length human *SNCA* cDNA subcloned into pCMV6-XL5 was purchased from Origene. Plasmid DNA was first diluted in OPTI-MEM I (Gibco-BRL, USA) and then mixed with FuGENE HD Transfection Reagent. Following a 20-minute incubation period at room temperature, the DNA-FuGENE HD Transfection Reagent mixture was added to the antibiotic-free transfection medium (DMEM containing glutamine). The cells were maintained in a 37°C incubator with a saturated humidity air atmosphere containing 5% CO_2_. The medium was replaced with normal culture medium 6 hours later. Cells were used for further experiments 48 hours after transfection.

### Transfection of siRNA

Small interfering RNA (siRNA) was Stealth select siRNA purchased from Invitrogen (Carlsbad, CA). The targeting sense sequence for human SNCA in SH-SY5Y cells is 5′-GACCAAAGAGCAAGUGACAAAUGUU-3′, and the one for rat SNCA in PC12 cells is 5′-GGGCGUCCUCUAUGUAGGUUCCAAA-3′. Stealth™ RNAi Negative Control Duplex (Medium GC, Invitrogen) was used as a negative control for the RNAi response. They were all transfected with Lipofectamine RNAi MAX (Invitrogen) according to the manufacturer's instructions. Cells were used for various experiments 48 hours after transfection.

### Isolation of mitochondria

We isolated mitochondria from three cell lines according to the method of Du et al. [49]. Mitochondria were isolated by centrifuging cell homogenates at 1,500 g for 5 min at 4°C. We adjusted the supernatant to 14% Percoll and recentrifuged at 12,000 g for 10 min. The supernatant was subjected to further centrifugation at 15,000 g for 20 min at 4°C, with the resultant supernatant being used as the cytosolic fraction. The loose mitochondrial pellet was resuspended and recentrifuged at 8000 g for 10 min. Highly purified mitochondria were used for immunoblotting assay.

### Apoptosis analysis

Detection of apoptosis by flow cytometry (FACScan; BD Company) was performed using the Annexin V-FITC apoptosis detection kit (BD Company) according to the manufacturer's instructions. A minimum of 10,000 cells were analyzed in each treatment.

### Western blotting analyses

Cells were collected after transfection for 48 hours. Cell lysates or mitochondrial lysates were prepared and ran on 10%–12% SDS-PAGE gels. Antibodies used were as follows: rabbit polyclonal anti-α-synuclein (1∶1,000, Cell signaling), rabbit antisera against COX IV (1∶1500, Cell signaling), mouse antibody against β-actin (1∶20,000, Sigma).

### Mitochondrial morphology assessment of fixed cells

Cells were plated in glass-bottom culture dishes for mitochondrial observation. After *SNCA* transfection for 48 hours, mitochondria were visualized by staining with MitoTracker-Red CMXRos (Molecular Probes) at a final concentration of 50 nM and cells were then processed for immunofluorescence as described below. Images of 20 cells from random view fields were taken from each group on UltraView VoX 3D live cell imaging system (PerkinElmer) using a 100× objective, Z-stacks were taken at 0.5 µm intervals, and the experiment was repeated three times. Cell images were analyzed for lengths of major and minor axes of the ellipse equivalent to the mitochondrion using Volocity 5.0 (Improvision, Lexington, MA) software. From these values, we calculated mitochondrial length (or aspect ratio, the ratio between the major and minor axes of the ellipse equivalent to the mitochondrion) [25], [26], [27]. Mitochondrial aspect ratio has a minimal value of 1 when it is a perfect circle and the value elevates when mitochondria become elongated.

### Mitochondrial morphology assessment of live cells

SH-SY5Y or PC12 cells were plated in glass-bottom culture dishes for mitochondrial observation on live cell imaging system. Mitochondria were visualized by staining with MitoTracker-Green FM or MitoTracker-Red CMXRos (Molecular Probes), both at a final concentration of 50 nM, or by transfection with a mitochondria-targeted GFP (Molecular Probes) according to the manufacturer's instructions. Images were taken and analyzed and mitochondrial length was quantified as described above.

### Immunofluorescence

Cells were rinsed with pre-warmed PBS (pH 7.2) and fixed in 4% paraformaldehyde for 30 min. After washed with PBS for 3 times, cells were permeabilized with 0.2% Triton X-100 for 30 min, and incubated with 10% fetal calf serum for 90 min. To determine α-synuclein-immunoreactive cells in culture, cells were sequentially incubated with rabbit polyclonal anti-α-synuclein primary antibody (Millipore) for 2 hours and FTIC-conjugated anti-rabbit IgG (Rockland) for 90 min, and washed with PBS between stages. Each step above was carried out at room temperature.

### Statistical analyses

The data are expressed as means ± SE and were subjected to statistical analysis via one-way ANOVA or non-paired, two-tailed Student's t-test. The level of statistical significance was set at P<0.05.

## Supporting Information

Figure S1 **Transfection efficiency of human α-synuclein in SH-SY5Y cells.** Different ratios of plasmid DNA (µg)/transfection reagent (µl) (1∶3, 1∶4, 1∶5, 1∶6) were applied to SH-SY5Y cells to optimize transfection efficiency. Immunoblotting analysis after transfection for 48 h showed that 1∶4, 1∶5 and 1∶6 of plasmid DNA/transfection reagent provided the best transfection efficiency for SH-SY5Y cells. *P<0.05, **P<0.01 compared with control.
(TIF)

Figure S2 **Mitochondrial preparation confirmation.** Cytosolic and mitochondrial extracts were both examined by immunoblotting for the presence of cytochrome oxidase subunit IV (COX IV) and β-actin. Representative immunoblotting image shows that mitochondrial fraction was enriched with mitochondrial marker COX IV, while few mitochondria were detected in cytosolic fraction.
(TIF)

Figure S3 **α-Synuclein overexpression promotes its mitochondrial localization in PC12 cells.** Further amplified images of Figure 4A demonstrate that α-synuclein overexpression increases its mitochondrial localization in PC12 cells.
(TIF)

Figure S4 **α-Synuclein knockdown prevents MPP^+^-induced cell apoptosis and mitochondrial fragmentation in PC12 cells.** **A.** Representative images taken by live cell imaging system show no obvious alteration in mitochondrial morphology between Neg group and RNAi group. MPP^+^ (1 mM) induces severe mitochondrial fragmentation in Neg group but has little effect on mitochondrial morphology in RNAi group. Scale bar for 10 µm. **B.** Immunoblotting assay demonstrates that α-synuclein expression is remarkably suppressed in PC12 cells transfected with *SNCA* siRNA (RNAi group) for 2–5 d, yet it is hardly affected in cells transfected with a negative control sequence (Neg group) (n = 5). **C.** Flow cytometric analysis of cell apoptosis shows that MPP^+^ leads to severe cell injury in Neg group, while it slightly harms PC12 cells in RNAi group (n = 4). **D.** Quantitative analysis of changes in mitochondrial length shows that MPP^+^ reduces mitochondrial length in Neg group, whereas it has little effect on the index in RNAi group. Images of 20 cells from each group were processed for mitochondrial morphology analysis, and the experiment was repeated three times. *P<0.05, **P<0.01 Neg versus RNAi; #P<0.05, ##P<0.01 compared with Neg control.
(TIF)
