# Supplementary figures and images for: Role of Alpha-Synuclein Protein Levels in Mitochondrial Morphology and Cell Survival in Cell Lines
Source: PLoS One. 2012 Apr 27;7(4):e36377. doi: 10.1371/journal.pone.0036377 (PMC3338674; doi:10.1371/journal.pone.0036377)

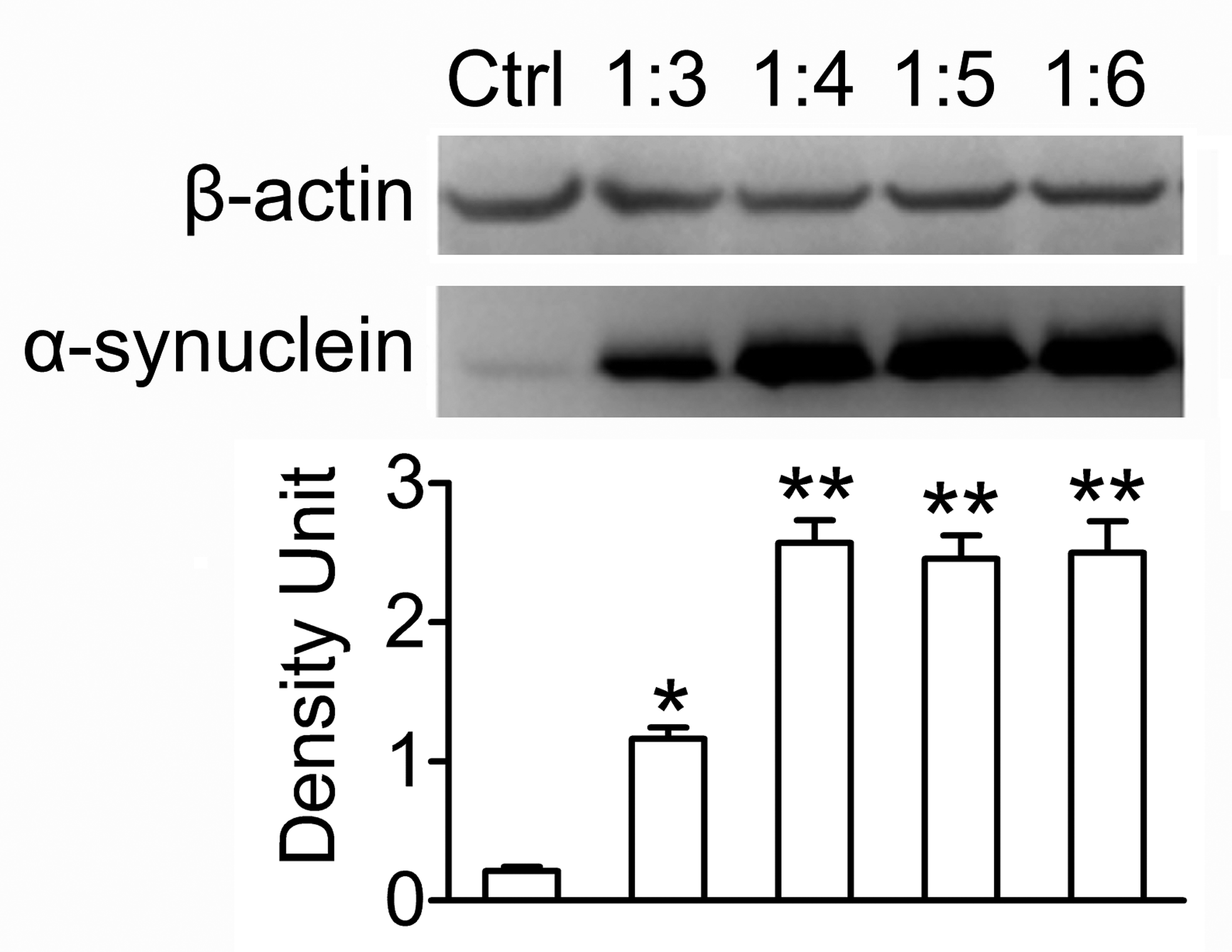

Supplement: Figure S1 — Transfection efficiency of human α-synuclein in SH-SY5Y cells. Different ratios of plasmid DNA (µg)/transfection reagent (µl) (1∶3, 1∶4, 1∶5, 1∶6) were applied to SH-SY5Y cells to optimize transfection efficiency. Immunoblotting analysis after transfection for 48 h showed that 1∶4, 1∶5 and 1∶6 of plasmid DNA/transfection reagent provided the best transfection efficiency for SH-SY5Y cells. *P<0.05, **P<0.01 compared with control. (TIF) [file pone.0036377.s001.tif]

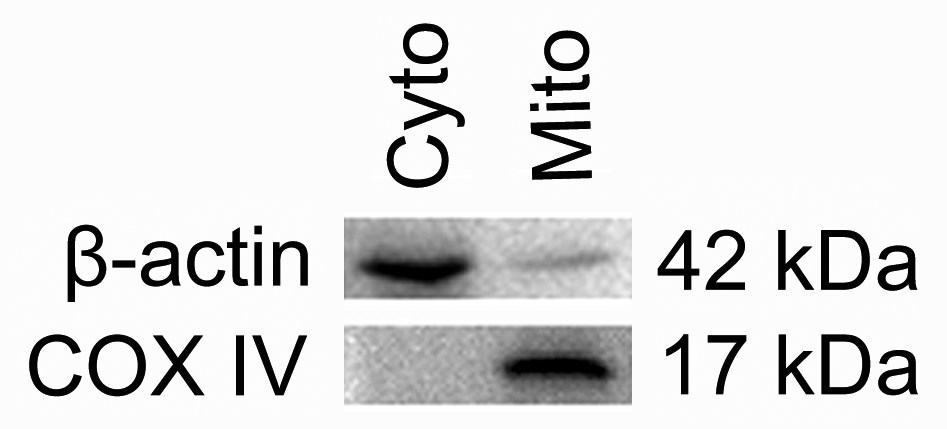

Supplement: Figure S2 — Mitochondrial preparation confirmation. Cytosolic and mitochondrial extracts were both examined by immunoblotting for the presence of cytochrome oxidase subunit IV (COX IV) and β-actin. Representative immunoblotting image shows that mitochondrial fraction was enriched with mitochondrial marker COX IV, while few mitochondria were detected in cytosolic fraction. (TIF) [file pone.0036377.s002.tif]

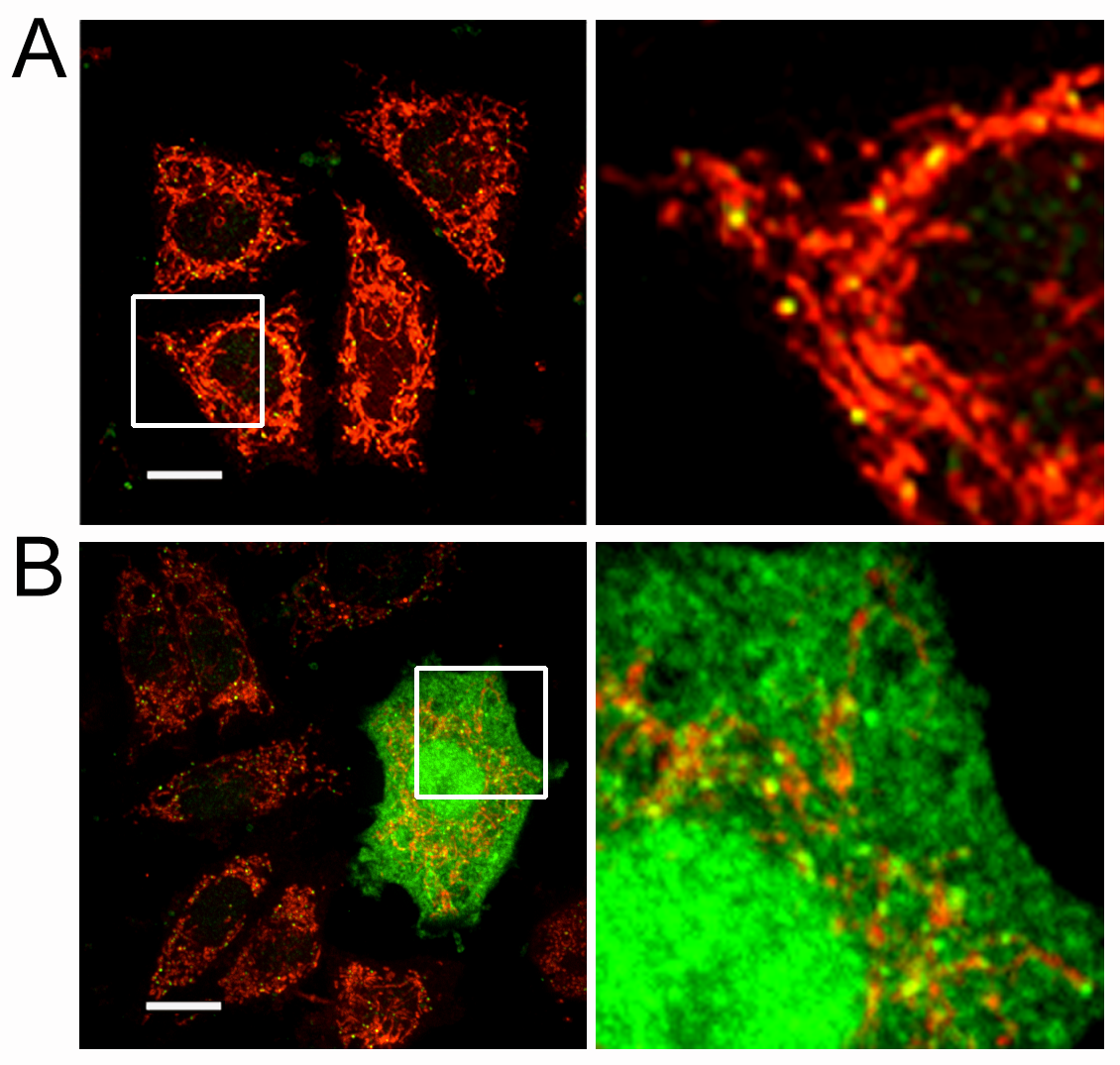

Supplement: Figure S3 — α-Synuclein overexpression promotes its mitochondrial localization in PC12 cells. Further amplified images of Figure 4A demonstrate that α-synuclein overexpression increases its mitochondrial localization in PC12 cells. (TIF) [file pone.0036377.s003.tif]

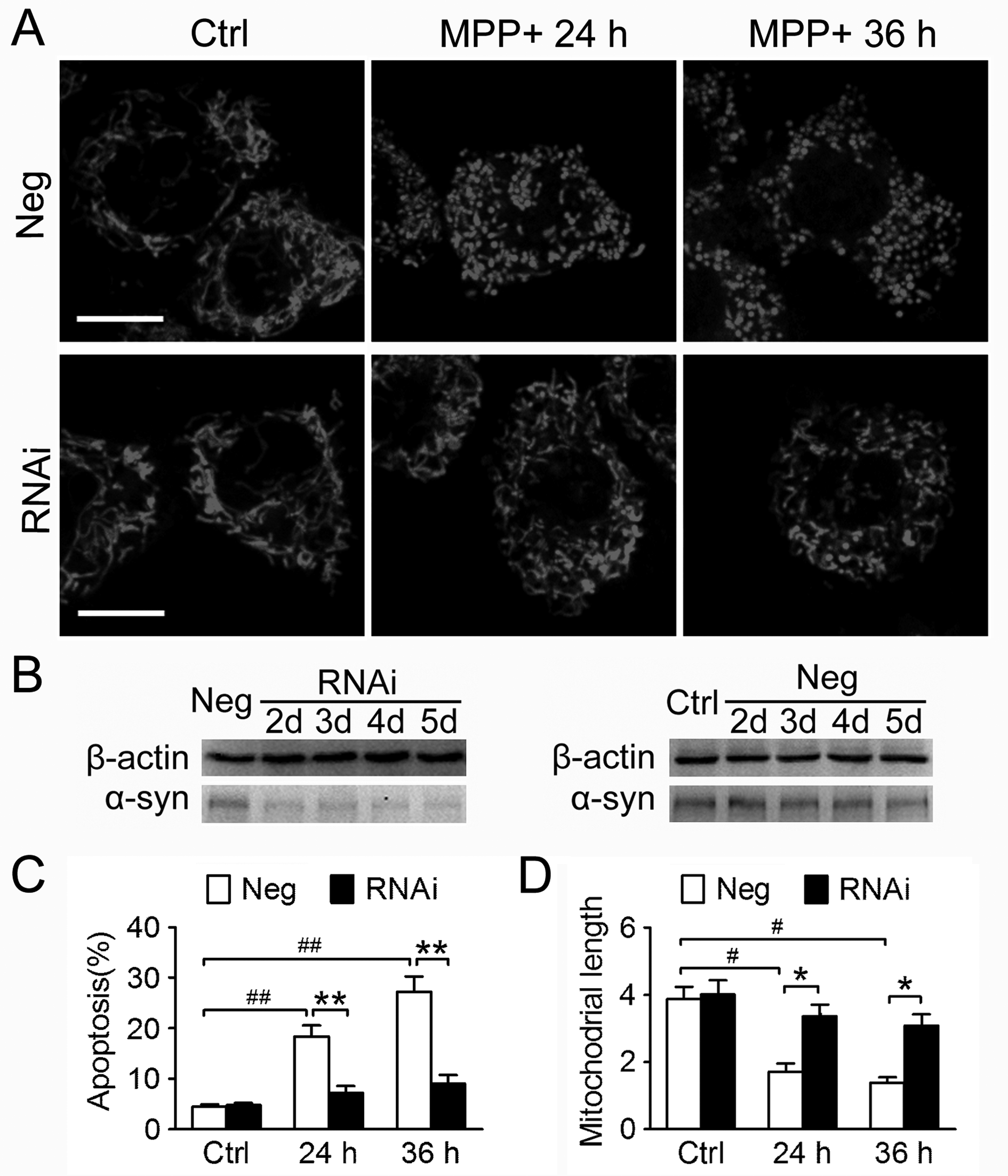

Supplement: Figure S4 — α-Synuclein knockdown prevents MPP+-induced cell apoptosis and mitochondrial fragmentation in PC12 cells. A. Representative images taken by live cell imaging system show no obvious alteration in mitochondrial morphology between Neg group and RNAi group. MPP+ (1 mM) induces severe mitochondrial fragmentation in Neg group but has little effect on mitochondrial morphology in RNAi group. Scale bar for 10 µm. B. Immunoblotting assay demonstrates that α-synuclein expression is remarkably suppressed in PC12 cells transfected with SNCA siRNA (RNAi group) for 2–5 d, yet it is hardly affected in cells transfected with a negative control sequence (Neg group) (n = 5). C. Flow cytometric analysis of cell apoptosis shows that MPP+ leads to severe cell injury in Neg group, while it slightly harms PC12 cells in RNAi group (n = 4). D. Quantitative analysis of changes in mitochondrial length shows that MPP+ reduces mitochondrial length in Neg group, whereas it has little effect on the index in RNAi group. Images of 20 cells from each group were processed for mitochondrial morphology analysis, and the experiment was repeated three times. *P<0.05, **P<0.01 Neg versus RNAi; #P<0.05, ##P<0.01 compared with Neg control. (TIF) [file pone.0036377.s004.tif]
